# Ophthalmic emergency-room visits during the Covid-19 pandemic – a comparative study

**DOI:** 10.1186/s12886-021-01976-6

**Published:** 2021-05-12

**Authors:** Veronika Yehezkeli, Ygal Rotenstreich, Liron Naftali Ben Haim, Ifat Sher, Asaf Achiron, Avner Belkin

**Affiliations:** 1 Department of Ophthalmology, Meir Medical Center, Kfar Saba, Israel; 2 The Sackler Faculty of Medicine, Tel Aviv University, Tel Aviv, Israel; 3 Goldschleger Eye Institute, Sheba Medical Center, Ramat Gan, Israel; 4 Bristol Eye Hospital, University Hospitals Bristol NHS Foundation Trust, Bristol, UK

**Keywords:** Covid − 19, Ophthalmic emergency, Visits, Comparisons, ER

## Abstract

**Background:**

The goal of this study is to compare ophthalmic emergency room (OER) visits during the Coronavirus disease-19 (Covid-19) pandemic to those during a control period.

**Methods:**

We compared all visits to the OER to Meir Medical Center in Israel, from March 15th to April 15th, 2020, during the Covid-19 pandemic and government mandated quarantine, to the same period in 2019. Factors analyzed were patient demographics, chief complaints, referral patterns, exam findings, treatments given, hospitalizations and surgical interventions.

**Results:**

We included in this study 1311 visits of 1158 patients, 477 during the 2020 Covid-19 pandemic and 834 during the same period in 2019. The demographic distribution (age, gender, and ethnicity) was similar between the two periods. LogMAR visual acuity at presentation was worse during the Covid-19 pandemic (0.42 ± 0.6 and 0.34 ± 0.5 in 2020 and 2019 respectively; *p* = 0.025) and the number of emergent surgeries was higher (3.7% in 2020 vs 1.8% in 2019, *p* = 0.026). In 2019 there was a higher likelihood of involvement of both segments of the eye (4.82% versus 1.2%, *p* < 0.01) and more diagnoses were given to each patient (1 ± 0.5 versus 0.93 ± 0.35, *p* = 0.001; During the Covid − 19 pandemic medications (both topical and systemic) were prescribed more often (1.22 ± 0.95 in 2020 and 0.84 ± 0.67 in 2019, *p* < 0.001).

**Conclusions:**

OER visits were less frequent during the Covid − 19 pandemic as compared to 2019, though the demographics of the patients remained unchanged. Visits during the pandemic tended to be for more severe ocular conditions, with worse visual acuity at presentation and required more medical and surgical treatment which imply higher necessity of ocular evaluation. This analysis can aid healthcare resource management in similar scenarios in the future.

## What was known

1. Visits to the Ophthalmic emergency room (OER) are unplanned events, the nature of which depend on living patterns that were dramatically affected by the Covid − 19 pandemic.

## What this paper adds

1. OER visits during the Covid − 19 pandemic tended to be for more severe ocular conditions and required more testing as well as more medical and surgical treatment.
2. As compared to the previous year, the demographics of the patients remained unchanged.

## Background

The Coronavirus disease-19 (Covid − 19) pandemic has had a significant effect on health care delivery in a wide variety of medical disciplines [1–3]. These effects were due to the different allocation of resources within the health care system, and different patterns of health care consumption. Between March 15th and April 15th, 2020, like in many parts of the world, Israel was under government mandated quarantine and people were instructed to limit their outings from home to a minimum. During this time, public hospitals in Israel were re-organized to prepare for the possible increase in Covid − 19 related morbidity. In Ophthalmology departments, several changes were made. First, all non-urgent testing and surgeries were cancelled. Second, clinic volumes were lowered significantly, and the number of on-site ophthalmologists was reduced. Third, tele-medicine was introduced to allow for continuation of care without frontal doctor-patient meetings whenever possible. Importantly, during March 2020 emergency regulations were issued and included, among others, prohibition of staying in public spaces and strict limitations on public transport. Since age was at that point a known risk factor for a severe disease and disease complications, the ministry of health recommended extra caution be taken by patients above 65 years old. Of course, medical service was always available to all patients regardless of their age.

Visits to the Ophthalmic emergency room (OER) are unplanned events, the nature of which depend on several factors. These factors include living patterns that were dramatically affected by the Covid − 19 pandemic, and the prevalence of diseases in the population [4]. The purpose of this study was to evaluate the nature of visits to the OER, to assess the care given in this setting during the Covid − 19 pandemic, and to compare these to a control period. We believe this analysis can aid healthcare resource management in means of anticipating, preparing, and mobilizing resources to improve and mitigate treatment and morbidity.

## Materials and methods

### Participants

This retrospective study was approved by the Institutional Review Board of the Meir Medical Center. Meir Medical Center is the 7th largest hospital complex in Israel, serving a population of approximately 750,000 people. We reviewed the files of consecutive OER visits between March 15th and April 15th, 2020 which was during the Covid − 19 pandemic, and during the same time period in 2019. All OER visits were included in the study, and none were excluded.

### Data collection

Data was recorded from the electronic medical record (EMR) of the OER visits. Collected parameters included patient demographics (age, gender and ethnicity), chief complaints, visual acuity, main clinical findings and management (auxiliary tests, medications prescribed, hospitalization and surgery). For the purposes of statistical analysis the Logarithm of the Minimum Angle of Resolution) LogMAR (equivalent for counting fingers was 1.85, hand motion was 2.3, light perception was 2.8, and no light perception was 2.9 [5]. In all cases in which the complaint was bilateral visual loss, the acuity recorded was that of the right eye. The results remained unchanged when using the average acuity of both eyes or the worse acuity of the two for analysis instead.

Distance from hospital was calculated using the nearest route suggested by Google Maps. Referral patterns were classified as self-referred, referred from a non-ophthalmologist physician, referred from a primary care ophthalmologist or a follow up from a prior visit to our clinic. Non-urgent visits were defined by minimal effect on visual acuity and mild discomfort [6].

### Statistical analysis

Data was analyzed with the SPSS software for windows version 20.0 by IBM. For the analysis of continuous data Student’s t-test was used for normally distributed variables and Kruskal-Wallis for non-parametric variables. For the analysis of categorical variables, Chi-Square or Fishers’ exact test were used as appropriate. In all analyses a two-sided *P* value < 0.05 was considered statistically significant. All presented means are accompanied by their respective standard deviations.

## Results

### Patient demographics

Overall, 1311 visits of 1158 patients were included in this study, 477 in 2020 during the Covid − 19 pandemic and 834 during the same period in 2019. Patients from both time periods were similar in all demographic characteristics (Table 1). Average age was 48.1 ± 20.1 years and 48.9 ± 21.4 years old in 2020 and 2019 respectively, and the majority were male in both time periods (60.58% in 2020 and 56% in 2019).

**Table 1:** Demographic characteristics of study participants

Variable	March–April 2019	March–April 2020	*p* Value
Number of eyes	834	477	
Age, years mean (SD)	48.8 (21.36)	48.1 (20.1)	*p* = 0.54
Male n (%)^a^	467 (56%)	289 (60.58%)	*p* = 0.1
Ethnicity n (%)			*p* = 0.96
Jewish	694 (83.2%)	397 (83.2%)	
Arab	130 (15.5%)	75 (15.7%)	
Other^b^	10 (1.1%)	5 (1%)	
Distance from hospital, Km			
Mean (SD)	14.8 (16.7)	19.2 (26.3)	***P*** **= 0.005**
Median (IQR)	11.9 (17.3)	13.5 (16.9)	

Abbreviations: *IQR* interquartile range, *SD* standard deviation, *Km* kilometer. ^a^ Percentage calculated from total visits

^b^Foreign workers from China, Nepal, Thailand; Tourists from Europe

**Fig. 1:**
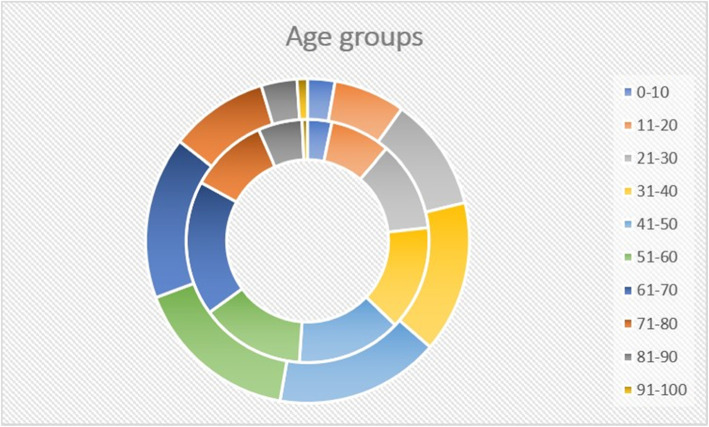
The distribution of patients by age; Inner circle 2019, outer circle 2020. No statistical difference was found between the age groups

On average, patients drove further from their home to the OER in 2020: 19.2 ± 26.3 km versus 14.8 ± 16.7 (*p* < 0.001). This was significant even when patients who came from over 100 km were excluded. A sub-analysis of distance travelled between Jewish and Arab patients revealed a longer mean distance for Arab patients which was not statistically significant between ethnicities for both time periods (2019: 14.53 ± 17.09 for Jews, 17.67 ± 16.12 for Arabs; 2020: 18.03 ± 27.23 for Jews, 25.09 ± 26.94 for Arabs; *P* > 0.05). The distribution of patients by age is presented in Fig. 1**.**

### Visit characteristics

**Fig. 2:**
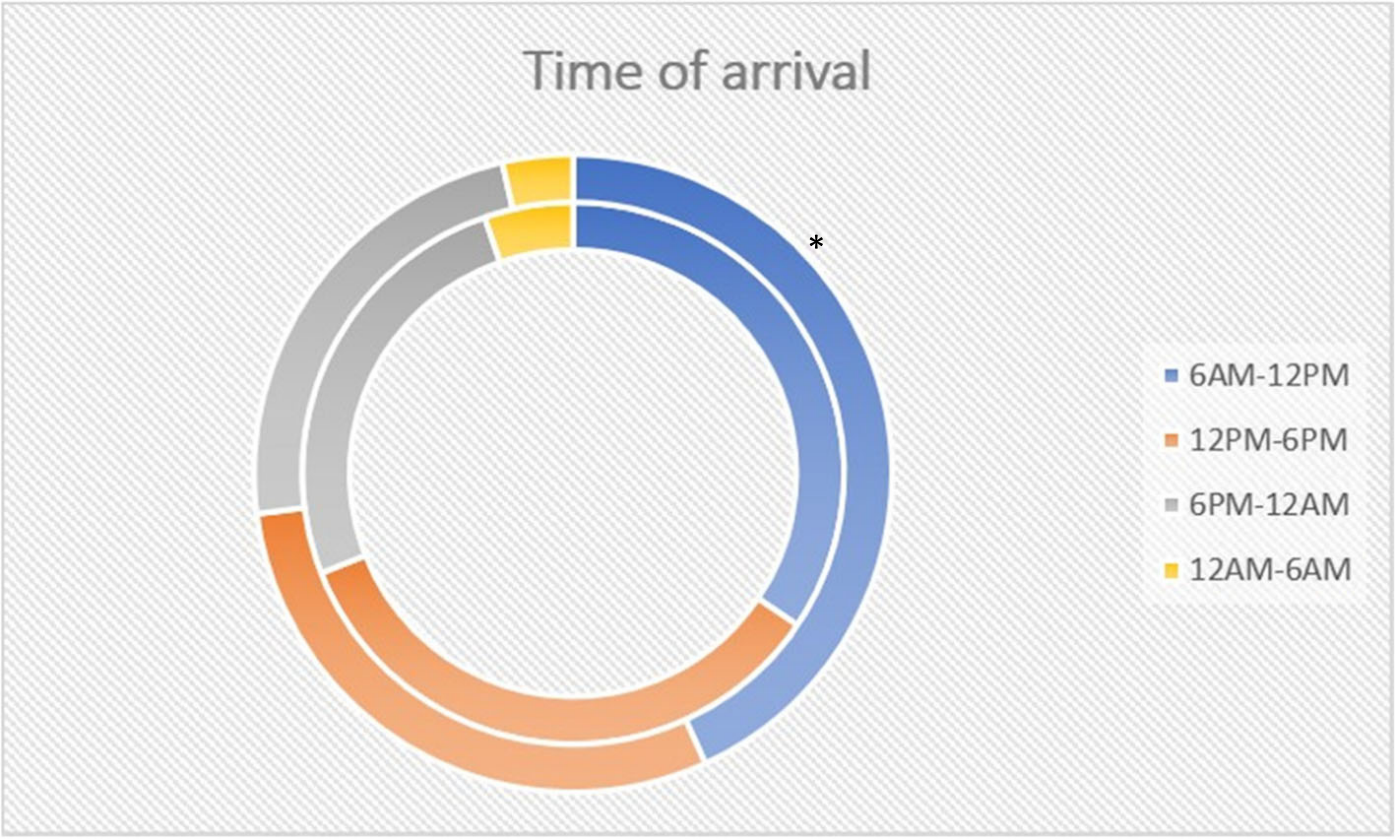
The time of day patients arrived at the emergency department; Inner circle 2019, outer circle 2020. Patients were more likely to arrive in the morning hours (6 AM-12 PM) in 2020 (*P* = 0.002*)

The proportion of follow-up visits was higher (*p* < 0.001) in 2020 as compared to 2019. The time of day patients arrived at the emergency department differed between the two time periods (Fig. 2) as patients were more likely to arrive in the morning hours in 2020 (*p* = 0.002). The time from onset of symptoms to the visit was similar between the periods. Table 2 shows visit characteristics for both groups.

**Table 2:** Visit characteristics

Variable	March–April 2019	March–April 2020	*P* Value
Referral n (%)			
Optometrist	1 (0.12%)	0 (0%)	
Primary care ophthalmologist	111 (13.3%)	63 (13.2%)	*p* = 0.958
Primary care non-ophthalmologist	232 (27.8%)	98 (20.5%)	***p*** **= 0.004**
In hospital consult	68 (8.1%)	26 (5.4%)	*p* = 0.068
Self-referral	381 (45.7%)	196 (41.1%)	*p* = 0.126
Follow up to prior visit at our clinic	41 (4.9%)	94 (19.7%)	***p*** **< 0.001**
Trauma n (%)			
No trauma	676 (81%)	405 (85%)	***p*** **= 0.001**
Indoor trauma	52 (6.2%)	15 (3.1%)	***p*** **= 0.015**
Outdoor trauma	106 (12.7%)	56 (11.7%)	*p* = 0.608
Eye n (%)			*p* = 0.163
OD/OS	701 (85.5%)	420 (88.2%)	
OU	119 (14.5%)	56 (11.7%)	
Complaint duration, days mean (SD)	3.9 (7.8)	4.5 (7.8)	*p* = 0.22
Chief complaint n (%)			
Reduction in vision monocular	83 (9.9%)	77 (16.1%)	***p*** **= 0.001**
Reduction in vision binocular	18 (2.2%)	8 (1.7%)	*p* = 0.682
Diplopia	13 (1.6%)	2 (0.4%)	*p* = 0.102
Floater	87 (10.4%)	26 (5.4%)	***p*** **= 0.002**
Other visual disturbance^a^	18 (2.1%)	27 (5.6%)	***p*** **= 0.001**
Lid related	64 (7.7%)	23 (4.8%)	***p*** **= 0.045**
Ocular pain monocular	395 (47%)	228 (47.8%)	*p* = 0.895
Ocular pain binocular	46 (5.52%)	18 (3.8%)	*p* = 0.183
Red eye	71 (8.5%)	31 (6.5%)	*p* = 0.188
Other^b^	56 (7%)	52 (11%)	***p*** **= 0.008**

Abbreviations *SD* standard deviation

^a^Metamorphopsia, “Clouds”, black bubbles, flashes of light

^b^Asymptomatic patients who were referred by other physicians to r/o papilledema, uveitis, high IOP, suspected Neovascular Age related Macular Degeneration, suspected Branch retinal vein occlusion and new rubeosis iridis

### Ophthalmologic examination and clinical findings

In 2020, visual acuity at presentation in the eye which was the reason for the visit was worse than in 2019 (0.42 ± 0.6 logMAR (20/52) vs. 0.34 ± 0.5 logMAR (20/43),*p* = 0.025) and there was a higher chance of involvement of both segments compared to 2019 (4.82% versus 1.2%, *p* < 0.001). Clinical findings in both time periods are shown in Table 3.

**Table 3:** Ophthalmologic examination and clinical findings

Variable	March–April 2019	March–April 2020	*P* Value
LogMar VA mean (SD)			
CC eye	0.34 (0.5)	0.42 (0.6)	***p*** **= 0.025**
Fellow eye	0.26 (0.4)	0.23 (0.4)	*p* = 0.23
IOP, mmHg mean (SD)			
OD	13.94 (4.6)	14.8 (5.7)	*p* = 0.065
OS	14.14 (4)	13.96 (5.9)	*p* = 0.683
Eye segment involved n (%)			
Anterior chamber	572 (68.6%)	319 (66.9%)	*p* = 0.524
Posterior segment	159 (19%)	91 (19%)	*p* = 0.995
Both	10 (1.2%)	23 (4.8%)	***p*** **< 0.001**
None^a^	93 (11.1%)	43 (9%)	*p* = 0.372
Main clinical finding n (%)			
Normal examination	85 (10.2%)	45 (9.4%)	***p*** **=** 0.659
Foreign body	108 (12.9%)	74 (15.5%)	*p* = 0.196
Conjunctivitis	72 (8.6%)	24 (5%)	***p*** **= 0.016**
Retinal Vascular event^b^	11 (1.3%)	9 (1.9%)	*p* = 0.484
Retinal tear	24 (2.9%)	13 (2.7%)	*p* = 0.873
Retinal Detachment	10 (1.1%)	8 (1.7%)	*p* = 0.474
Perforation	3 (0.3%)	0	
Neuro ophthalmology	19 (2.3%)^c^	16 (3.3%)^d^	*p* = 0.245
Anterior segment inflammation	23 (2.7%)	14 (2.9%)_	*p* = 0.852
Posterior segment inflammation	1 (0.1%)	0	
Glaucoma (open and closed angle)	12 (1.4%)	19 (4%)	***p*** **= 0.004**
Dry eye	37 (4.4%)	17 (3.6%)	*p* = 0.444
Corneal laceration	85 (10.2%)	54 (11.3%)	*p* = 0.523
Conjunctival laceration	13 (1.5%)	8 (1.7%)	*p* = 0.87
SPK	66 (7.9%)	30 (6.3%)	*p* = 0.277
Chalazion	14 (1.7%)	9 (1.9%)	*p* = 0.828
PVD	62 (7.4%)	21 (4.4%)	***p*** **= 0.03**
Lid swelling	43 (5.1%)	22 (4.6%)	*p* = 0.663
SCH	31 (3.7%)	14 (2.9%)	*p* = 0.53
Corneal abscess	36 (4.3%)	40 (8.4%)	***p*** **= 0.002**
Blepharitis	21 (2.5%)	6 (1.2%)	*p* = 0.157
Retinopathy^e^	10 (1.2%)	10 (2.1%)	*p* = 0.242
VH	14 (1.7%)	13 (2.7%)	*p* = 0.227
Other^f^	59 (7.1%)	58 (12.1%)	*p* = **0.002**
Number of diagnoses mean (SD)	0.93 (0.35)	1.0 (0.5)	***p*** **= 0.001**
One diagnosis n (%)	724 (86.8%)	388 (81.3%)	***p*** **= 0.008**
Two diagnoses and more n (%)	25 (3%)	43 (9%)	***p*** **< 0.001**

Abbreviations: *SPK* superficial punctate keratitis, *PVD* posterior vitreous detachment, *SCH* subconjunctival hemorrhage, *VH* vitreous hemorrhage, *SD* standard deviation

^a^Eyelids related or neuro-ophthalmologic complains which didn’t refer to eye segments (i.e. visual migraine, nerve palsies, etc. …)

^b^Arterial occlusions and Vein occlusions

^c^6th nerve palsy (3), 3rd nerve palsy (2), Anterior ischemic optic neuropathy (AION) (2), Optic neuritis (ON) (2), visual migraine (3), anisocoria (1), benign cyclic mydriasis (1), papilledema (5)

^d^6th nerve palsy (3), ON (7), papilledema (2), bell’s palsy (1), compressive optic neuropathy (1), optic atrophy (1), 3rd nerve palsy (1)

^e^Diabetic retinopathy / hypertensive retinopathy / high-altitude retinopathy

^f^Neovascular age related macular degeneration, corneal graft rejection, choroidal neovascularization, lattice degeneration, corneal opacity, subluxation of the lens, commotio retina, hyphema, dacryocystitis

In 2020, more diagnoses were given to each patient – 43 (9%) patients in 2020 and 25 (3%) patients in 2019 received two or more diagnoses (*p* < 0.001, Table 3). Patients were given one of 24 different Ophthalmic diagnoses. The chief complaint was bilateral in 11.7% (*n* = 56) and 14.5% (*n* = 119) in 2020 and 2019, respectively (*P* = 0.195). Conjunctivitis and posterior vitreous detachment (PVD) were significantly less likely to occur in 2020 as compared to 2019 (5% vs.8.6%, *p* = 0.016 for conjunctivitis and 4.4% vs. 7.4%, *p* = 0.03 for PVD). A diagnosis of glaucoma (both open and closed angle) (4% vs. 1.4%, *p* = 0.04) and corneal abscess (8.4% vs. 4.3%, *p* < 0.001) were more common in 2020 as compared to 2019. The proportion of visits resulting from trauma was lower in 2020 (72 (15%) in 2020 and 167 (19%), *p* = 0.001). This was true when indoor trauma was assessed separately as well (*p* = 0.015). Visits due to floaters (*p* = 0.002) and lid related complaints (*p* = 0.045) were more likely to occur in 2019.

We performed a sub-analysis of “serious” diagnoses, for which the authors felt the visit to the OER was necessary and justified. The following diagnoses were considered” serious” - retinal vascular event, retinal detachment, perforation, anterior segment inflammation, posterior segment inflammation, glaucoma, corneal abscess and vitreous hemorrhage. The cumulative percent of these diagnoses was 12.9% in 2019 and 21.6% in 2020, this difference is statistically significant (*P* = 0.00).

### Case management

Table 4 shows management patterns in both periods. In 2020, the percentage of patients who were discharged without receiving any treatment was lower than in 2019 (23.26% versus 29.25%, respectively, *p* = 0.003). Specifically, topical antibiotics, topical steroids, systemic steroids, hypotensive drops, and artificial tears were all prescribed more in 2020 (*p* < 0.005 for all). Two or more medications were prescribed to 151 (31.6%) patients in 2020, and to 97 (11.6%) patients in 2019 (*p* < 0.001).

**Table 4:** Case Management

Variable	March–April 2019	March–April 2020	*P* Value
Treatment n (%)			
No treatment	244 (29.2%)	104 (23.2%)	***p*** **= 0.003**
Topical antibiotics	331 (39.7%)	227 (47.6%)	***p*** **= 0.005**
Topical steroids	88 (10.5%)	97 (20.3%)	***p*** **< 0.001**
Lubrication	187 (22.2%)	143 (30%)	***p*** **= 0.003**
Systemic antibiotics	47 (5.6%)	22 (4.6%)	*p* = 0.425
Systemic steroids	4 (0.47%)	10 (2.1%)	***p*** **= 0.01**
Intravitreal injections	19 (2.3%)	12 (2.5%)	*p* = 0.851
Glaucoma treatment (Topical and systemic)	14 (1.7%)	28 (5.9%)	***p*** **< 0.001**
Other^a^	12 (1.4%)	41 (8.6%)	***p*** **< 0.001**
Number of treatments mean (SD)	0.84 (0.67)	1.22 (0.95)	***p*** **< 0.001**
One treatment n (%)	493 (59.1%)	221 (46.3%)	***p*** **< 0.001**
Two or more treatments n (%)	97 (11.6%)	151 (31.6%)	***p*** **< 0.001**
Auxiliary tests n (%)			
No Auxiliary tests	751 (90%)	388 (81.3%)	***p*** **< 0.001**
OCT	64 (7.7%)	62 (12.3%)	***p*** **= 0.002**
Visual fields	20 (2.4%)	16 (3.3%)	*p* = 0.38
FA	6 (0.7%)	13 (2.7%)	***p*** **= 0.006**
US	8 (0.9%)	14 (2.9%)	***p*** **= 0.012**
Number of Auxiliary tests mean (SD)	1.02 (0.16)	1.06 (0.3)	***p*** **< 0.001**
Office procedures n (%)			
No office procedures	673 (80.7%)	368 (77.1%)	*p* = 0.127
Foreign body removal cornea	63 (7.5%)	58 (12.2%)	***p*** **= 0.008**
Foreign body removal conjunctiva	47 (5.6%)	17 (3.6%)	*p* = 0.094
Laser Barrage	22 (2.6%)	15 (3.1%)	*p* = 0.594
Eye irrigation	19 (2.3%)	6 (1.2%)	*p* = 0.311
Hospitalizations n (%)	28 (3.3%)^b^	9 (1.9%)^c^	*p* = 0.165
Follow up location n (%)			
Hospital	254 (30.4%)	222 (46.5%)	***p*** **< 0.001**
Outside clinic	403 (48.3%)	169 (35.4%)	***p*** **< 0.001**
None	177 (21.2%)	85 (17.8%)	*p* = 0.154
Number of emergent surgeries	15 (1.8%)	17 (3.7%)	***p*** **= 0.046**
Time from diagnosis to surgery, days mean (SD)	2.86 (1.91)	2.23 (1.95)	*p* = 0.364
Number of in hospital consults	16 (1.9%)	14 (2.9%)	*p* = 0.236

Abbreviations: *OCT* optical coherence tomography, *FA* fluorescein angiography, *US* ultrasound, *SD* standard deviation

^a^Acyclovir; Anti-allergic treatment (topical and systemic)

^b^ 3rd nerve palsy, optic neuritis (ON) and anisocoria in neurologic department (3), corneal abscess (1), peri-orbital cellulitis (3), high IOP (5), ruptured globe and conjunctival laceration (2), endophthalmitis following anti- VEGF injection, retinal artery occlusion (RAO) and vein occlusion for systemic evaluation (3), evisceration (1), retinal detachment (RD) (3) and thyroid eye disease for systemic steroids (1)

^c^RAO (1), retinitis (1), RD (1), ON (4), high IOP and VZV with corneal involvement

Auxiliary testing was performed more frequently in 2020 as compared to 2019. Statistically significant differences were found in the use of optical coherence tomography (OCT, 12.3% vs 7.7%, *p* = 0.002), fluorescein angiography (FA, 2.7% vs 0.7%, *p* = 0.006) and Ultrasound B-scan (2.9% vs 0.9%, *p* = 0.012). Removal of corneal foreign body was more likely in 2020 (12.2% vs 7.5%, *p* = 0.008), while other slit lamp procedures were performed at similar rates in both time periods.

There was no difference in the number of hospitalizations - 9 (1.9%) in 2020 and 28 (3.3%) in 2019, *p* = 0.165). In 2019 three patients were hospitalized in the neurology department with 3rd nerve palsy, Optic neuritis (ON) and anisocoria. Twenty five patients were hospitalized in the ophthalmology department with corneal abscess, peri-orbital cellulitis, high IOP, ruptured globe and conjunctival laceration, endophthalmitis following anti-vascular endothelial growth factor (VEGF) injection, retinal artery occlusion (RAO) and vein occlusion for systemic evaluation, evisceration, retinal detachment (RD) and thyroid eye disease for systemic steroids.

All hospitalizations in 2020 were in the ophthalmology department with diagnoses of RAO, retinitis, RD, ON, high IOP and Varicella Zoster Virus (VZV) with corneal involvement.

Follow up visits were more likely to be scheduled for the hospital clinic in 2020, as oppose to a referral back to the community (46.5% vs 30.4%, *p* < 0.001).

The number of emergent surgeries was higher in 2020 [3.7% (*n* = 17) vs. 1.8% (*n* = 15), *p* = 0.026]. In 2019 we performed twelve vitrectomies, one lensectomy, one evisceration and one repair of a lacerated eyelid. In 2020 we performed eleven vitrectomies, three lensectomies and three glaucoma surgeries. The significant difference between the two periods was in the urgent glaucoma surgeries.

## Discussion

The aim of this study was to characterize visits to the OER during the Covid − 19 pandemic when the country was under government-mandated quarantine, and to assess its effect on management strategies to aid healthcare resource management.

We found that the number of OER visits during the Covid − 19 pandemic was reduced by 43% as compared to the same month the previous year. Since non-urgent visits make up to three quarters of OER visits in normal times [7], the reduction in visit numbers during the pandemic is likely due to patient’s higher threshold for seeking medical care. Moon et al. also found a 32% decrease in the total volume of OER visits in 2020 compared to prior years [8]. Moreover, Posarelli et al. showed a significant reduction of visits during the lockdown, compared with those of the pre-lockdown period (reduction of 65.4%) and with those of the same period in 2019 (reduction of 74.3%) [9]. It should be noted that sight-threatening emergencies do occur in patients who are reluctant to leave the house and seek care. For these patients and for at-risk populations in general, measures should be implemented to allow for phone-based triage and telemedicine. Although the number of visits was significantly reduced during the pandemic, the age of the patients remained unchanged. This finding was unexpected in the context of the Covid − 19 pandemic, as the strictest limitations were upon patients over the age of 65 since morbidity and mortality from the virus was higher in this group. Rehman et al. from Chandigarh, India, on the contrary, showed an average of younger age of presentation during lockdown, reinforcing the hypothesis that the elderly preferred staying at home [10].

Although females account for roughly half of the population, our study showed a lower incidence of ocular emergencies in this population. This tendency was reported by other studies on ocular emergencies as well. In a study conducted on patients admitted to the ophthalmic emergency room in Cluj-Napoca, Romania, 28% of patients were female [11] whereas in a similar study from Ankara, Turkey, a reported 30.6% of the patients were female [12]. This difference may reflect behavior less prone to trauma, cultural differences, or differences in access to health care.

On average, patients travelled a longer distance on average to arrive at the OER during the Covid − 19 pandemic as compared to the previous year in this study. This finding remained significant when patients who came from over 100 km were excluded. This is in keeping with the finding of a higher rate of serious ocular conditions in 2020.

On 31 December,2019, Israel’s population was estimated at 9,136,000 residents of whom 74.1% were Jews, 21.0% were Arabs [13]. Our study participants were 15.5% of Arab ethnicity compared to 83.2% of Jewish ethnicity. A sub-analysis of distance travelled between Jewish and Arab patients revealed a non-significant longer mean distance for Arabs. This difference may reflect the difference in the distribution of Jewish and Arab settlements as it pertains to distance from our medical center, social and cultural differences, as well as differences in access to health care.

Visits to the OER during the Covid − 19 pandemic were due to more serious medical issues as compared to visits from the previous year. This was evident by a worse visual acuity at presentation, a higher rate of monocular reduction in vision as the presenting symptom, a higher rate of involvement of both segments of the eye, a higher rate of emergent surgeries, a higher rate of multiple diagnoses and a higher rate of “serious” diagnoses. This finding is likely due to a higher threshold for seeking ophthalmic care. Also, topical antibiotics, topical and systemic steroids and hypotensive drops were all prescribed more in 2020 as compared to 2019.

A study from Taiwan [14] found that VA could be an indicator for determining the priority and time of ocular emergencies requiring ophthalmic intervention in patients visiting the ED for eye-related reasons. A LogMAR VA score of 0.45 (decimal equivalent of 0.4) had the highest discrimination power for identifying whether a patient needed ophthalmology intervention or admission to an ophthalmology ward. Our results of VA in 2020 (LogMAR 0.42) correspond to the score that Kang, E.Y., et al. found to correlate with more severe ophthalmic emergencies [14]. Our results are consistent with those of Moon et al. and Posarelli et al- minor and non-urgent visits decreased, and there was a resultant 9% increase in the proportion of primary diagnoses considered urgent, respectively [8, 9].

OER visits in 2020 were less likely to be related to trauma as compared to 2019. This can be explained by the lifestyle modifications made necessary by the quarantine. There was no difference in outdoor trauma in our study. The lower incidence of trauma related visits during the Covid − 19 pandemic in our study is consistent with the recent report by Pellegrini et al. from Italy [15]. However, Pellegrini et al. did find an increase in indoor trauma which interestingly, was not found in our study, despite the home quarantine.

We found that the time from onset of symptoms to the OER visit did not differ between the two periods. OER visits were significantly more likely to occur in the morning in 2020, and the rate of nighttime visits was reduced. This is probably a reflection of altered working schedules due to the quarantine.

Wu et al. [16] reported that one third of Covid − 19 positive patients had ocular manifestations consistent with conjunctivitis. Interestingly, Gangaputra et al. [17] reported that among patients tested for Covid − 19, red eye and epiphora were significantly more common in the Covid − 19 negative group. Our cohort of ambulatory patients during 2020 were less likely to be diagnosed with conjunctivitis. This is possibly due to the community-based spread of this disease, which is likely blunted by social distancing and quarantine. Diagnoses which have been linked to stress (like central serous chorio-retinopathy or blepharitis and chalazion, [18–20]) were not more likely to occur in 2020 in our study.

Different epidemiological reports tried to characterize the magnitude and patterns of visits to the OER. Their results are comparable to our 2019 findings. Docherty et al. [21] analyzed data from emergency ophthalmology referrals in 2017 and found that PVD (12.2%) was the most common diagnosis, followed by corneal abrasion (7.4%) and retinal detachment (RD) (5.3%). Our results from 2019 showed 7.4, 10.2, 1.1% of PVD, corneal abrasion and RD respectively. The differences are minor and are likely explained by different referral patterns between the studies.

Contrary to our finding that RD rate was similar in 2019 and 2020, Wickham et al. showed that the number of patients presenting with RD fell significantly following introduction of isolation measures (an average drop of 62% compared to 2019). These observations have also been made by vitreoretinal surgeons in departments across the UK [22]. This finding, suggesting that the fear of vision loss was outweighed by the fear of seeking hospital care during the pandemic was not found in our study.

Channa et at. reported corneal abrasions (13.7%) and foreign body in the external eye (7.5%) as the leading diagnoses in the emergent category, and conjunctivitis (28.0%), subconjunctival hemorrhages (SCH) (3.0%), and styes (3.8%) were the leading diagnoses in the non-emergent category in 2015 in the United States [23]. In 2019, we found comparable rates of foreign bodies (12.9%), corneal abrasions (10.2%), SCH (3.7%) and Chalazion (1.7%).

We found that OCT, FA and ultrasound B-scan were all performed more frequently in 2020 as compared to 2019. The location of follow-up in 2020 was more likely to be in the hospital, and not in the outside clinic. These findings are likely related to the higher rate of serious ocular conditions, to the limited availability of community-based eye care during the pandemic, or alternately to the tendency of the treating physician to try and avoid follow up visits as much as possible. There were 17 Surgeries in 2020, and 15 in 2019. This corresponds to the higher threshold of patients to arrive with non-urgent issues. However, it is important to note that visits to the OER in 2020 were more likely to result in surgery as compared to the year before.

This study has several limitations: First, medical care was given by at least 10 different Ophthalmologists working in our facility, though this was true for both time periods. However, it expresses the real-life working patterns. Second, is the retrospective nature of the study with its inherent limitations in data collection and interpretation. Third is the limited duration of testing, with 1 month possibly underrepresenting some less common diagnoses. Fourth, since less patients arrived during 2020, some of the statistically significant comparisons were based on percentage and not the absolute numbers. Since the demographic distribution was similar between the years, we believe that this limitation is part of the conclusions of our study. Fifth, we cannot analyze how many of the patients in the OER were Covid-positive since there was no routine testing.

In summary, OER visits during the pandemic tended to be for more severe ocular conditions and required more testing as well as more medical and surgical treatment. While there were less frequent visits during the Covid − 19 pandemic. as compared to the previous year, the demographics of the patients remained unchanged. This data should be considered when planning for future scenarios that share similarities to this one.

## Data Availability

The datasets used and/or analysed during the current study are available from the corresponding author on reasonable request.
